# The effect of background music on episodic memory and autonomic responses: listening to emotionally touching music enhances facial memory capacity

**DOI:** 10.1038/srep15219

**Published:** 2015-10-15

**Authors:** C.A. Alice Mado Proverbio, Valentina Lozano Nasi, Laura Alessandra Arcari, Francesco De Benedetto, Matteo Guardamagna, Martina Gazzola, Alberto Zani

**Affiliations:** 1 Milan-Mi Center for Neuroscience, Dept. of Psychology, University of Milano-Bicocca Piazza dell’Ateneo Nuovo 1 Milan, 20126, Italy; 2 IBFM-CNR, Via Fratelli Cervi, Milan, 20090, Italy

## Abstract

The aim of this study was to investigate how background auditory processing can affect other perceptual and cognitive processes as a function of stimulus content, style and emotional nature. Previous studies have offered contrasting evidence, and it has been recently shown that listening to music negatively affected concurrent mental processing in the elderly but not in young adults. To further investigate this matter, the effect of listening to music vs. listening to the sound of rain or silence was examined by administering an old/new face memory task (involving 448 unknown faces) to a group of 54 non-musician university students. Heart rate and diastolic and systolic blood pressure were measured during an explicit face study session that was followed by a memory test. The results indicated that more efficient and faster recall of faces occurred under conditions of silence or when participants were listening to emotionally touching music. Whereas auditory background (e.g., rain or joyful music) interfered with memory encoding, listening to emotionally touching music improved memory and significantly increased heart rate. It is hypothesized that touching music is able to modify the visual perception of faces by binding facial properties with auditory and emotionally charged information (music), which may therefore result in deeper memory encoding.

- A total of 54 non-musicians listened to joyful or emotionally touching music, rain sounds, or silence while studying hundreds of faces
- Heart rate and diastolic and systolic blood pressure were measured during face encoding
- Except for emotionally touching music, auditory background interfered with memory recall
- Touching music is able to bind visual properties with emotionally charged information (music), which results in enhanced memory

The effects of listening to background music on concurrent mental processing are controversial. Overall, although listening to music appears to have positive effects on emotions and especially on motor behavior (such as athletic performance), it appears to interfere with reading and memory tasks (see ref. 1 for a review). In automobile driving, listening to music appears to alleviate driver stress and reduce aggression; however, in conditions that require attention and mental concentration, driving performance is impaired^2^.

Two perspectives have been proposed to account for the effects of background music on cognitive processes: the *Cognitive-Capacity model* and the *Arousal-Mood hypothesis*. Kahneman’s capacity model^3^ postulates that only a limited pool of resources is available for cognitive processing at any given moment. When concurrent tasks compete for limited resources and their combined demands exceed the available capacity, *capacity interference* occurs. Only a portion of the task information is processed and therefore performance deteriorates. The interference caused by task-irrelevant information (for example, listening to music) also depends on the complexity of the information that is being processed and on the workload that is required to process task-relevant information. Indeed, increasingly complex musical distractions may result in decreased cognitive performance^4^.

In contrast, the *Arousal-Mood* hypothesis posits that listening to music affects task performance by positively influencing arousal and mood^5^, which is a phenomenon that is also known as the Mozart effect^6^. This hypothesis has been supported by several studies that have investigated the effect of listening to background music on the performance of cognitive tasks. For example, improvements in verbal memory encoding^7^, autobiographical memory in Alzheimer patients^8^, verbal and visual processing speed^9^, arithmetic skill^10^, reading^11^, and second language learning^12^ have been documented.

Conversely, reduced performance in the presence of background music has also been demonstrated (for example, see ref. 5). As noted by Kämpfe and colleagues^1^ in an excellent meta-analysis, background music may have a small but persistent negative effect on memory performance-related tasks, such as memorizing advertisements (e.g., ref. 13), memorizing nonsense syllables or words (especially in the presence of loud music)^14^,^15^, remembering previously read texts and reading performance^16^. Listening to background music vs. silence has also been reported to interfere with many additional cognitive processes, including the ability to perform arithmetic^17^; performance on verbal, numerical and diagrammatic analysis tests^18^,^19^; multimedia learning^20^; the learning of new procedures^21^; reading^22^,^23^,^24^; and inhibition of performance of the Stroop task^25^.

Recently, Bottiroli *et al.*^26^ found that listening to Mozart (as compared to silence and white noise) improved declarative memory tasks in the elderly. They interpreted these data in the context of the so-called “arousal and mood hypothesis”^5^ because performance systematically increased under conditions that induced positive mood and arousal. In contrast, Reaves *et al.*^27^ indicated that listening to music comes at a cost to concurrent cognitive functioning. In their study, both young and old adults listened to music or to silence while simultaneously studying face-name pairs. The participants’ abilities to remember the pairs were then tested as they listened to either the same or to different music. The results showed that older adults remembered 10% fewer names when listening to background music or to rain compared to silence. Therefore, although music may help to relax individuals who are trying to concentrate, it appears that it does not help them to remember what they are focusing on (new information), especially as they age.

Overall, the data are conflicting, although it appears that listening to background music most interferes with tasks that involve memory, especially for verbal items. To the best of our knowledge, the effect of listening to music on the ability to remember nonverbal or linguistic items has not been previously investigated.

To further investigate this matter, in this study, the ability to remember human faces was evaluated in the context of different types of acoustic background, including silence (as a non-interfering control), the sounds of rain and storms (generally thought to have a relaxing effect^28^,^29^), and occidental music of different emotional content and style. A previous study^30^ compared listening to silence with listening to music or rain during a backward digit span task and found no effect of auditory background on performance. To provide information about the effect of background noise on alertness and arousal levels, as well as possible autonomic correlates of emotional responses, heart rate and systolic and diastolic blood pressure were measured during the first part of the experiment (study phase). In this session, 300 unknown male and female faces were presented to participants in an explicit memory encoding situation. The study session was followed by a memory test that consisted of evaluating the recognition of 200 previously viewed faces that were randomly interspersed with 100 new faces, under conditions of silence. Hit and error percentages were quantified as functions of the experimental conditions (listening to emotionally touching music, to joyful music, to the sound of rain, to silence).

The aim of the present study was to determine the autonomic and cognitive correlates of non-verbal memory processing as a function of the nature of an auditory background (or the lack of it, i.e., silence). It was hypothesized that music would either increase arousal levels and therefore improve memory, as predicted by the Mozart effect^5^,^6^, or that it would interfere with memory by overloading attentional systems and therefore reduce subjects’ performance of the memory task^4^. The effect imparted by the emotional content of music was also explored by comparing the condition of listening to joyful music with that of listening to emotionally touching (sad) music. With respect to the effects of listening to music on autonomic parameters, it appears that although listening to music might reduce anxiety and induce mental relaxation under certain experimental conditions or clinical settings, it has little or no influence on hemodynamic parameters, except for a tendency to increase systolic blood pressure^31^,^32^,^33^.

In this study autonomic measures were recorded in non-musician controls since we aimed at investigating the effect of auditory background on perceptual processing in individuals not particularly specialized in music processing. Indeed, it is known that the musicians’ brain reacts differently from that of other individuals to auditory information of various nature, including phonologic stimuli, noise and sounds^34^. Although not a specific research aim, the effect of participants’ sex in memory for face (as a function of auditory background) was also investigated since some literature has shown gender effects on episodic memory and musical emotion processing. Indeed evidences have been provided of a female greater advantage in episodic memory tasks for emotional stimuli^35^ and of females’ hypersensitivity to aversive musical stimuli^36^.

## Materials and Methods

### Participants

Fifty-four healthy participants (27 males and 27 females), ranging in age between 18 and 28 years (mean age = 22.277 years), were included in this study. They were all right-handed with normal hearing and vision and none had suffered from previous or current psychiatric or neurological diseases. All participants received academic credits for their participation and provided written consent. The experiment was performed in accordance with the relevant guidelines and regulations of and was approved by the Ethical Committee of the University of Milano-Bicocca. The participants were blinded to the purpose of the experiment. None of the participants were musicians, and none of them had ever studied music or played a musical instrument, or had a musical activity as a hobby or specific interest. This information was specifically ascertained through the administration of a detailed questionnaire.

### Stimuli

#### Visual stimuli

A total if 448 colored pictures of anonymous human faces of women (N = 224) and men (N = 224) of different ages were used as visual stimuli. The faces were selected from available, open access, license-free databases. They were equally represented by sex and age ranges (children, adolescents, young adults [25–35 years], mature adults [35–60 years] and the elderly). The pictures only showed a given subject's face up to the base of the neck. The image size was 265 × 332 pixels. The characters wore various accessories (e.g., glasses, hats, earrings, etc.) and depicted various emotional expressions, ranging from joy to anger, all of which were matched across stimulus categories. The valence and arousal of each face was assessed in a preliminary validation study that was performed on 15 Psychology University students^37^ through the administration of a modified version of the *Self Assessment Manikin* (SAM)^38^ an affective rating system. In this system, a graphic figure depicting values along each of 2 dimensions of a continuously varying scale is used to indicate emotional reactions. Judges could select any of the 3 figures comprising each scale, which resulted in a 0–2 point rating scale for each dimension. Ratings were scored such that 2 represented a high rating on each dimension (i.e., high arousal, positivity), and 0 represented a low rating on each dimension (i.e., low arousal, negativity), with 1 representing an intermediate score. On the basis of the valence and arousal ratings that were obtained from the validation study^37^, faces were randomly assigned to various auditory background conditions, which were also matched for sex and age of the persons depicted, so that the average valence and arousal of faces did not differ across blocks of stimulations. Stimuli were equiluminant, as ascertained by an ANOVA that was performed on individual luminance measurements that were obtained via a Minolta luminance meter.

#### Auditory stimuli

Pieces of music were selected on the basis of a validation that was performed on 20 orchestra directors, composers or teachers at various Italian Conservatories (18 men and 2 women), whose mean age varied between 50 and 60 years and who freely provided lists of the most emotionally touching classic instrumental music pieces from a tonal and atonal repertoire. Tonal music was defined as a musical production having a tonal center around which melody and harmony are based, including monodic productions from the Middle Ages. Atonal music was defined as a musical production (usually dated after 1910) that did not have a tonal center or that did not use multiple tonal centers simultaneously. After an initial selection, movie soundtracks, opera pieces and highly popular pieces of music were discarded. The selected list was then re-presented to the judges while asking them to choose the 3 most emotionally touching and 3 most joyful pieces (according to their own aesthetic preferences) for the tonal and atonal categories. The pieces with the highest ratings across the tonal and atonal repertoires were considered, and their similarities in structure, rhythm and in ensemble complement/instrumentation were also taken into account. In the end, the pieces that were voted to be used as stimuli in this study including the following:

- *Part, Arvo - Cantus in memoriam de Benjamin Britten (*atonal, touching)
- *Hindemith, Paul - First movement from I Kammermusik* (atonal, joyful)
- *Bach, Johann Sebastian - II movement from Concert in D minor for 2 violins (BWV 1043)* (tonal, touching)
- *Beethoven, Ludwig van - IV Movement of Symphony* (tonal, joyful)

Both rain sounds (obtained from an audio downloaded from Internet named “75 minutes of thunder and rain - relaxing noise for your ears” https://www.youtube.com/watch?v=WvRv-243Cmk&spfreload=10) and all musical excerpts were cut into 1-minute-long pieces, matched for intensity by means of *MP3Gain* software (89,0 dB) and faded at the end (last second) via *Audacity* software, and were therefore transformed into MP3 files. The modulation of tonality (used to provide variety) and its possible effect on autonomic parameters were not considered in this study.

### Procedure

**Figure 1:**
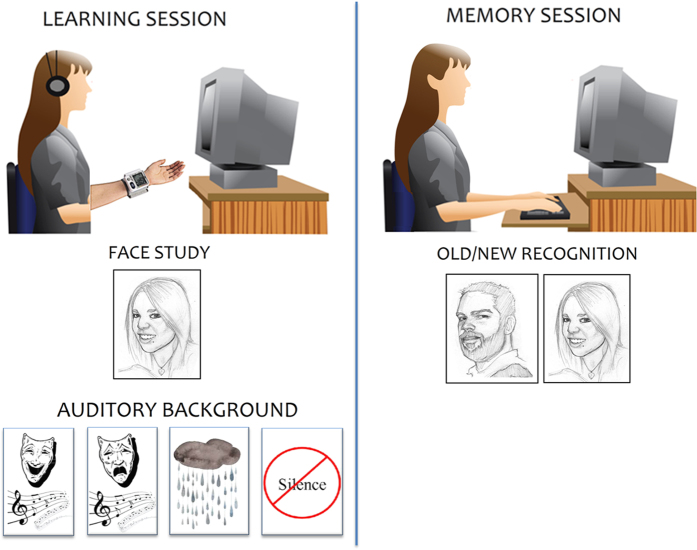
Schematic of the experimental paradigm, which included two sessions of face encoding and memory tasks. AMP and AZ contributed to the drawing of this figure.

The experiment consisted of two different sessions (see fig. 1 for a schematic of the paradigm). It was preceded by a training phase in which 56 unique pictures of women and men of various ages were presented in association with an auditory background. The subjects were instructed to pay attention to the faces that were presented in the 2 training sequences, which were followed by a short old/new discrimination task in which the left and right hands were alternated between when being used to respond. The first training session consisted of presenting 20 different faces to subjects as they listened to jazz music from “Freedom: an Instrumental approach to Jazz music” (n°12, *Wet atmosphere*, Julian Carl, JC Records, 2014). In the second training session, in which the opposite responding hand was used, an additional 20 faces were presented with an auditory background of natural sounds (ocean waves). In both sessions, subject wore headphones and wrist devises (on the left hand) that measured heart rate and blood pressure. The third training sequence consisted of the presentation of 8 old faces and 8 new faces that were randomly mixed. In this sequence, the subjects did not wear headphones or devises that measured autonomic responses. The auditory background was complete silence. The participants were instructed to respond as accurately and quickly as possible, using the index finger to indicate old faces and the middle finger to indicate new faces.

After subjects were acquainted with the task requirements and experimental settings, the experimental session started.

In the study or learning session, participants sat comfortably in front of a computer screen at a distance of 114 cm in an anechoic chamber under dimly lit conditions. A total of 300 faces were randomly presented at the center of the screen for 800 ms each with an ISI of 1300 ms. The stimuli were equally divided as a function of auditory background conditions (each auditory clip lasting 60 seconds) and matched across categories for sex, age, expression, valence and arousal. Stimulus delivery was performed using *Evoke software (Asa System)*. Subjects wore headphones and wrist devises that measured heart rate and blood pressure.

The session consisted in the presentation of 15 sequences separated by short pauses. The order of stimulus presentation was random and randomly varied across subjects. The wrist devices that measured heart rate and blood pressure was activated at the beginning of each sequence and stopped at the end. In this way it was assured a good timing of physiological responses to the cognitive stimuli.

During the memory test session, participants were presented with 300 faces (200 old and 100 new) in silent conditions and without wearing headphones or wrist devises for autonomic response measurements. The faces were presented for 800 ms each with an ISI of 1300 ms. The task requirements were the same as in the training memory task: old/new face discrimination with finger choice and response time and hits recording.

### Physiological recording

Data were not sampled but continuously acquired. Since each auditory fragment lasted 1 minute, both heart rate and blood pressure were acquired and averaged as per minute value and processed after the end of recording. Xanthine intake (i.e. caffeine) was controlled. All subjects were tested in the morning, and had no more than 1 breakfast coffee. None of them have been assuming medications affecting the SNC in the last 2 weeks. It was ascertained that no physical exercise was practiced by participants before the experiment, and participants were required to rest sitting down before the study for about 10 minutes to achieve a basal state. At this aim, 3 sequences of training were administered to all subjects before the beginning of the recording session.

### Data Analysis

The mean percentages of correct responses (arcsine transformed), response times (RTs in ms), and the mean values of heart rate and diastolic and systolic blood pressure that were measured during the learning session underwent five independent analyses of variance, with between group (sex, 2 levels: male, female) and within group (auditory background, 4 levels: joyful music, touching music, rain, silence) factors of variability.

A further analysis of variance was performed to compare the recognition rates of old and new faces that were measured during the test session. Between group (sex, 2 levels: male, female) and within group (face familiarity, 2 levels: old, new) factors of variability were included. Tukey’s test was used for post hoc comparisons of means.

## Results

**Figure 2:**
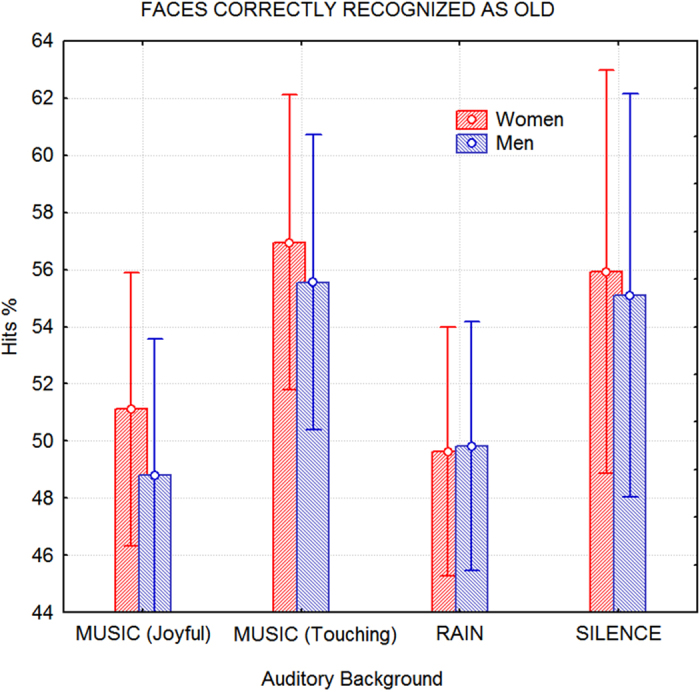
Hit percentages in the memory test as a function of auditory background during the study session. Nonverbal episodic memory recall was enhanced when study occurred either in silence or in the presence of emotionally touching music. Although women exhibited better performance on the test, especially while listening to music, the difference was not significant.

Figure 2 indicates the mean percentages of correct recognition of old faces (along with standard deviations) as a function of the sex of the viewers and the auditory background conditions. Although women tended to exhibit better performance on the task, sex was not a significant factor. ANOVA results indicated the significance of auditory background (F3,156 = 5.9; p < 0.0008), and higher percentages of correct facial recognition were obtained under auditory backgrounds comprised of emotionally touching music (vs. joyful music, p < 0.01; vs. rain, p < 0.007) or of silence (vs. joyful music, p < 0.03; vs. rain, p < 0.02) compared to other conditions.

**Figure 3:**
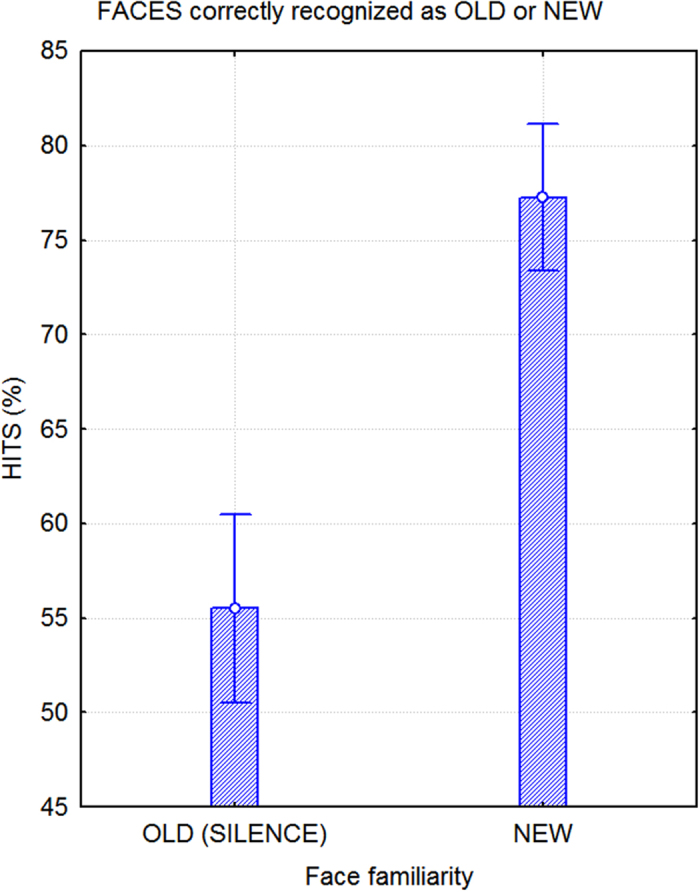
Hit percentages in the memory test. The data indicate that it was much easier for the participants to recognize new faces compared to old faces. Additionally, the participants were very successful at the task, despite the large number of faces that had to be remembered.

An ANOVA that was performed to compare hits of old versus new faces independent of auditory background showed a strong effect of stimulus familiarity (F1,52 = 33.3; p < 0.00001), which indicated that the test subjects had a much higher recognition of new faces (correctly rejected as unfamiliar) than old faces (correctly recognized as old), as displayed in Fig. 3. No sex differences in performance were observed.

**Figure 4:**
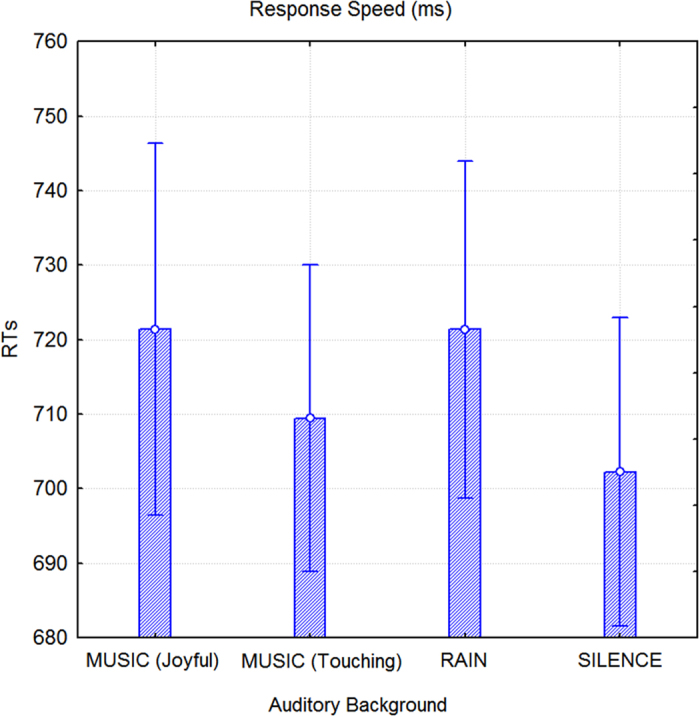
Mean RTs for correctly recognized old faces as a function of the auditory background present during the study session. Nonverbal episodic memory recall was significantly faster if study occurred in silent conditions or in conditions of emotionally touching music.

An ANOVA that was performed on RTs indicated the significance of auditory background (F3,156 = 5.1, p < 0.0022); test subjects exhibited faster RTs to faces that were studied in the presence of an auditory background of emotionally touching music (vs. joyful music, p < 0.04; vs. rain, p < 0.04) or silence (vs. joyful music, p < 0.001; vs. rain, p < 0.001) compared to other conditions. Figure 4 displays the mean RTs corresponding to correct recognition of old faces as a function of the presence of auditory background during learning.

**Figure 5:**
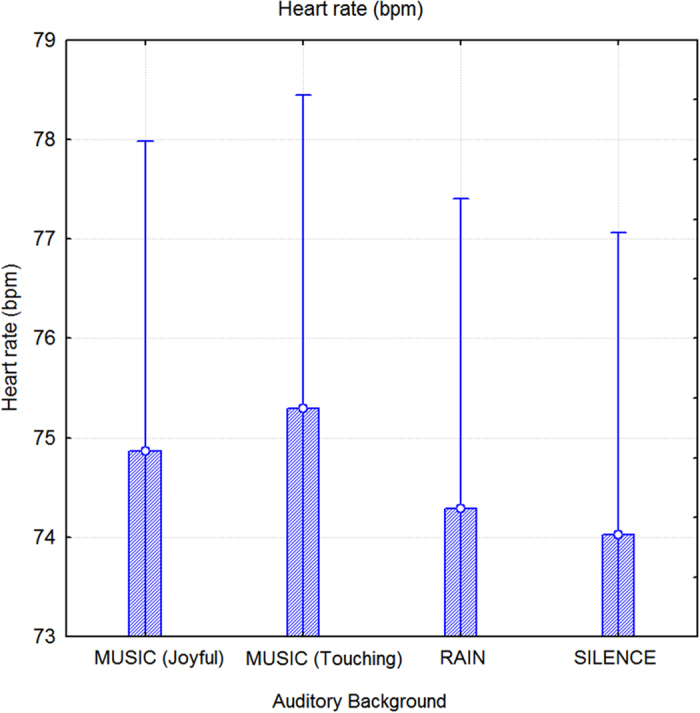
Mean heart rate (beats per minute) measurements recorded during different auditory background conditions. Participants exhibited significantly faster heart rates while listening to music (especially emotionally touching music) compared to rain sounds or silence. The intensity of auditory background (in dB) was matched across conditions and therefore the changes in heart rate possibly reflected increased cognitive and emotional processing.

An ANOVA that was performed to assess heart rate measurements indicated the significance of auditory background (F3,156 = 3.5; p < 0.018). Post hoc comparisons showed that the test subjects exhibited significantly faster heart rates while listening to emotionally touching music compared to rain (p < 0.026) or silence (p < 0.006); this was also true in the case of listening to emotionally touching versus joyful music (p < 0.06). Listening to joyful music also tended to enhance heart rate (p < 0.06) compared to silence, as displayed in Fig. 5.

**Figure 6:**
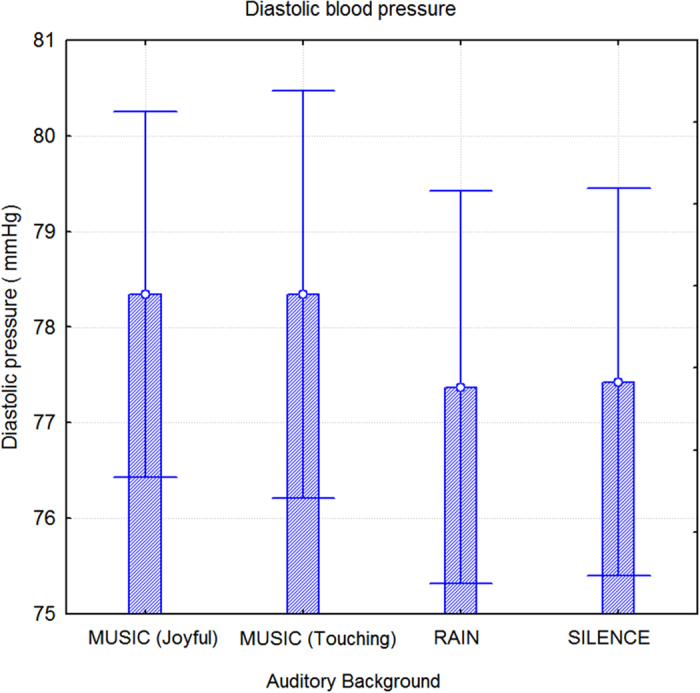
Diastolic (minim) blood pressure (diaBDP) values recorded as a function of auditory background. Music listening tended to increase diaBDP (p < 0.08).

A statistical analysis of diastolic blood pressure (diaBLP) measurements demonstrated an effect of sex (F1, 52 = 11.1; p < 0.0016), with lower diaBLP values in women (74.5 mmHg; SE 1.36) than men (8.1 mmHg; SE = 1.36). The effect of auditory background did not reach statistical significance (F3.156 = 0.08) but diaBLP tended to increase when subjects listened to emotionally touching (p < 0.07) and joyful (p < 0.08) music, as compared to sounds of rain or silence. The means and standard deviations corresponding to the above analyses are shown in Fig. 6.

**Figure 7:**
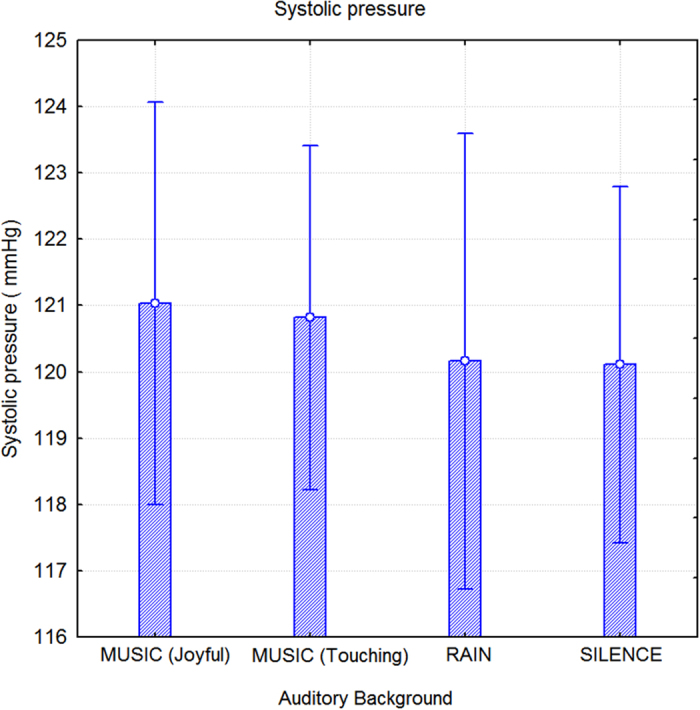
Systolic (maximal) blood pressure (sysBDP) values recorded as a function of auditory background. No effect was found between listening to music or rain sounds compared to silence.

An ANOVA that was performed on systolic blood pressure (sysBDP) measurements indicated a significant effect corresponding to sex (F1,52 = 32.3; p < 0.000001), with much lower sysBDP values in women (112.6 mmHg, SE = 1.96) than men (128.5 mmHg, SE = 1.96), and no significant effect caused by auditory background, as displayed in Fig. 7.

## Discussion

The aim of this study was to investigate how exposing subjects to varying auditory backgrounds while they engaged in a memory task affected later recognition performance. Response times were significantly faster and the recognition rate was higher for faces that were studied either in complete silence or in the presence of emotionally touching background music. Behavioral data demonstrated a higher recognition rate for new faces (correctly rejected as unfamiliar in 77.3% of hits with a 22.7% rate of error) than old faces (correctly recognized as familiar in 55.5% of hits with a 44.5% rate of error). This pattern is in accordance with previous literature, and it indicates that regardless of the large number of faces that were presented (N = 448), participants were able to accurately reject approximately 4 out of 5 new faces on the basis of a lack of familiarity. In a previously conducted electrophysiological study^39^, the recognition rate of 200 total faces was compared to that of 100 new faces and produced 79.3% correct hits for the former and 66.3% for the latter. Similarly, Yovel and Paller^40^ obtained hit rates of 87.8% for new faces and 65.3% for old faces. In the ERP study that was conducted by Currand and Hancock^41^ and included 360 faces, the memory recognition rates were approximately 90% for new faces and 81% for old faces. Considering that there were a greater number of stimuli in the present study, the performance was satisfactory, especially with respect to new faces. Overall, the recognition rate for old faces was a little bit lower in this than in other studies not featuring an interfering auditory background. Learning conditions were purposely made difficult to overload cognitive and perceptual systems and to determine whether the effects of listening to music and rain on visual learning were disruptive or enhancing.

Overall, the results of this study demonstrated that subjects more accurately encoded faces while listening to emotionally touching music compared to listening to rain or joyful music, similarly to conditions of silence. The most plausible explanation for this enhancement (or lack of interference) is that listening to emotionally touching music increased the arousal of the listeners, which was indicated by their increased heart rates. However, the arousal hypothesis does not hold true in this case per se because heart rates were also increased while listening to joyful music (which was associated with an increased number of facial recognition errors). Furthermore, listening to music generally tended to increase blood pressure compared to listening to rain sounds. The significant cost of listening to joyful music, which had the same intensity (in dB) as the emotionally touching music and rain sounds, must therefore not be interpreted as a lack of arousal activation but rather as lacking the beneficial effect that is imparted by musically-induced emotions on the ability to encode faces. Therefore, a hypothesis can be proposed that suggests that listening to emotionally touching music leads to emotionally-driven audiovisual encoding that strengthens memory engrams for faces that are visualized in this context, whereas listening to either rain or joyful music produces interfering effects by overloading perceptual channels during face encoding, as predicted by numerous studies that have described the persistent negative effects of listening to music on memory performance^1^,^4^,^13^,^14^,^15^,^17^,^18^,^19^,^20^,^21^,^22^,^23^,^24^,^25^. Indeed, according to Jancke^42^ (2008), “nostalgic music” has a strong influence on episodic memory. A recent study by Gold *et al.*^43^ investigated the effects of music on mnemonic capacity. In this study, music that was considered to be pleasant by subjects was contrasted with emotionally neutral music; both types of music were listened to by musician and non-musician subjects. During music listening, participants were engaged in encoding and later recalling Japanese ideograms. The results showed that subjects with musical expertise exhibited better performance on memory tasks while listening to neutral music, whereas subjects with no musical training (as in our study) more successfully memorized the studied ideograms while listening to emotionally touching music. These group differences might be interpreted by assuming that musicians dedicate more cognitive and attentional resources to the technical analysis of a preferred song and its musical properties. Conversely, the better performance at ideogram recall that was exhibited by non-musically trained participants as they listened to emotionally pleasant music might be due to increased attentional and arousal levels that were stimulated by the music. Indeed, numerous studies support the hypothesis that musical perception is able to modify how the brain processes visual information, which is the same principle that underlies the concept of the movie soundtrack^44^,^45^,^46^,^47^,^48^. In this case, music can strongly influence the interpretation of a film narrative by becoming integrated into the memory along with visual information and therefore it provides continuation, directs attention, induces mood, communicates meaning, cues memory, creates a sense of reality, and contributes to the aesthetic experience^44^. Furthermore, music can convey various types of emotional information via its harmony, rhythm, melody, timbre, and tonality, which can inspire multiple types of emotions in the listener, both simultaneously and in succession^49^.

The ability of emotional sounds to influence visual perception has been shown experimentally for stimuli such as complex IAPS (*International Affective Picture System*) scenes^50^,^51^, photographs of faces and landscapes^52^, emotional facial expressions^53^ and schematics of faces embedded in noise^54^. In particular, with regard to faces, it has been shown that subjects were more accurate at detecting sub-threshold happy faces while listening to happy music and vice versa for sad faces and sad music. This suggests that music is able to modulate visual perception by altering early visual cortex activity and sensory processing in a binding modality^54^. In a separate study^53^, participants rated photographs of crying, smiling, angry and yawning faces while concurrently being exposed to happy, angry, sad and calm music, or no music, and the results indicated that the participants made more favorable judgments about a crying face when listening to either sad or calm background music. Based on the current literature, it can be hypothesized that listening to music, especially emotionally touching music, might alter the visual perception of faces by making perceived faces (that were balanced for intensity and valence as uni-sensory visual stimuli) more emotionally charged and arousing to the viewer via a mechanism of audiovisual encoding. The higher arousal value of faces that were perceived in the presence of emotionally touching music (and to a lesser extent joyful music) was indicated by the increased heart rates of the participants that were measured under this condition. The result that higher hit rates were achieved with respect to emotive faces when participants were listening to emotionally touching music is compatible with current neuroscientific literature on facial memory. For example, untrustworthy faces are better remembered than trustworthy faces^55^; disgusting faces are better remembered than non-disgusting faces^56^; and faces expressing negative emotions are better remembered than neutral faces^57^,^58^. It is thought that this type of enhanced facial memory is due to a more general advantage that is imparted by remembering faces that are representative of negative or threatening contexts, and it is associated with increased activity in the amygdala, hippocampus, extrastriate, and frontal and parietal cortices during facial encoding^58^. A similar phenomenon might occur when faces are perceived in an arousing or emotional context (e.g., in a thriller movie with a scary soundtrack or in our study on listening to emotionally touching music). In others words, music might strengthen memory engrams by enhancing affective coding and enabling multimodal, redundant audiovisual memory encoding.

Although auditory background heavily affected memory accuracy and heart rate, it appeared to have little effect on blood pressure, except for a slight tendency to increase it during music listening. With regard to the effect of music listening on autonomic responses (blood pressure, heart rate, and respiratory rate), the literature is very conflicting. Although it has been shown that listening to music can reduce pain intensity and systolic blood pressure in patients during postoperative recovery^59^ and can reduce stress levels and heart rate in patients with coronary heart disease and cancer^60^, a reduction in heart rate or blood pressure caused by listening to music has not been demonstrated in healthy controls. For example, in a study conducted by Radstaak and colleagues^31^, healthy participants had to perform a mental arithmetic task while being exposed to harassment to induce stress. Afterward, participants were assigned to one of several “recovery” conditions in which they either (1) listened to self-chosen relaxing or happy music, listened to an audio book, or sat in silence. Systolic blood pressure, diastolic blood pressure, and heart rate were continuously monitored. The results indicated that although listening to both relaxing and happy music improved subjects moods, it did not diminish stress-enhanced systolic blood pressure. Therefore, mental relaxation was not associated with an improvement in autonomic parameters. In another interesting study^32^, systolic and diastolic blood pressure (BPsys, BPdia) were monitored as participants sat in silence and as they listened to 180-second-long recordings of two different “relaxing” and two different “aggressive” classical music excerpts. The results showed that listening to relaxing classical music and listening to aggressive classical music both increased BPsys, whereas autonomic modulation was lower under conditions of silence. Furthermore, in a study by Tan *et al.*^33^, the effect of relaxing music on heart rate recovery after exercise was investigated. Twenty-three healthy young volunteers underwent treadmill exercise and were then assessed for heart rate recovery and subjected to saliva analysis. The participants were either exposed to sedating music or to silence during the recovery period immediately following the exercise. No differences were found between exposure to music or silence with respect to heart rate recovery, resting pulse rate, or salivary cortisol. Overall, it appeared that although listening to music reduced anxiety under certain experimental settings, it did not seem to strongly influence hemodynamic parameters, except for a tendency to increase systolic blood pressure, which is consistent with the results of the present study.

In this study, accuracy and RT data indicated that participants committed more errors and were much slower when learning occurred under a background of rain sounds (vs. emotional music or silence). Although it is thought that listening to natural sounds (e.g., sounds of a rippling ocean, a small stream, soft wind, or a bird twittering) may produce relaxing and anxiety-reducing effects^61^, it has not been demonstrated that benefits to the learning process are imparted by listening to such sounds while studying and memory encoding. For example, a study that compared listening to silence versus listening to music or rain sounds during a backward digit span task found that auditory background produced no effect on performance whatsoever^30^. A study on the perception of white noise^62^, which shares several auditory properties with rain sounds (expect for artificiality), showed that listening to natural sounds (a running horse) and music tones decreased the ability of subjects to recall memories of scenes from their daily lives (compared to a condition of silence), whereas listening to white noise improved memory performance by improving connectivity between brain regions that are associated with visuospatial processing, memory and attention modulation. These results can be interpreted by assuming that the perception of recognizable and structured auditory objects (natural or musical sounds) interferes with memory processing, which is in agreement with the cognitive capacity model^4^. Conversely, listening to unstructured white noise does not produce such interference and alternatively increases cerebral arousal levels, in agreement with the arousal hypothesis^5^. In this context, the rain sounds and types of music that were used in the present investigation overloaded the perceptual systems of the participants, as shown by their reduced levels of performance on the assigned tasks compared to a condition of silence. However, listening to emotionally touching music benefitted concurrent emotional processing (associated with significantly increased heart rate), in agreement with a study conducted by Gold *et al.*^43^ and Quarto *et al.*^63^ In support of this hypothesis, several studies have provided evidence that listening to pleasant or emotionally arousing music can increase the heart rate of the listener^64^,^65^,^66^. Overall, the data indicate that perception of emotionally touching music can modify visual perception by binding visual inputs with emotionally charged musical information, resulting in deeper memory encoding.

One of the possible study’s limits is the existence of a culturally-mediated difference in the aesthetic musical preference between the judges and naïve participants who listened to the selected pieces. Indeed, while music evaluation resulting in the “touching and “joyful” characterization was performed by professional musicians that, as a result of their specific profession, have developed a quite positive aesthetic preference for classical music, naïve subjects (selected on the basis of their limited interest in music of whatever style) might have potentially found it boring or not interesting. Although aesthetics is based on liking or not an artwork, we assumed that touching and joyful music excerpts carried in their compositional structure some universal properties able to affect auditory processing of people not particularly skilled in music processing. The data strongly support this initial assumption, that music aesthetical preference is not only culturally, but also biologically based.

## Additional Information

**How to cite this article**: Proverbio, A. M. *et al.* The effect of background music on episodic memory and autonomic responses: listening to emotionally touching music enhances facial memory capacity. *Sci. Rep.* **5**, 15219; doi: 10.1038/srep15219 (2015).
