# Erratum: The effect of background music on episodic memory and autonomic responses: listening to emotionally touching music enhances facial memory capacity

**DOI:** 10.1038/srep17237

**Published:** 2015-12-14

**Authors:** Alice Mado Proverbio, Valentina Lozano Nasi, Laura Alessandra Arcari, Francesco De Benedetto, Matteo Guardamagna, Martina Gazzola, Alberto Zani

Scientific Reports 5: Article number: 1521910.1038/srep15219; published online: 10152015; updated: 12142015

The original version of this Article contained typographical errors in the spelling of the author Alice Mado Proverbio which was incorrectly given as C.A. Alice Mado Proverbio. This has now been corrected in the PDF and HTML versions of the Article.

